# The Treatment of Certain Functional and Organic Affections of the Nervous System

**Published:** 1866-09

**Authors:** C. E. Brown-Sequard

**Affiliations:** New Yokk


### SELECTED ARTICLES.

THE TREATMENT OF

### CERTAIN FUNCTIONAL AND ORGANIC AFFECTIONS OF THE NERVOUS SYSTEM,

BEING REMARKS MADE BY INVITATION BEFORE THE AMERICAN MEDICAL ASSOCIATION AT THE LATE MEETING HELD IN BALTIMORE,

By PROF. C. E. BROWN-SEQUARD, M. D., F. R. S., ETC., OF NEW YORK.

I propose, gentlemen, to offer a few remarks which shall bear more particularly upon certain improvements which have been made within the last twenty or thirty years in the treatment of nervous affections. In introducing this subject I can only refer to the great influence which a proper knowledge of the reflex phenomena has exerted upon an accurate appreciation of the causes which are at work in producing both functional and organic affections. I will not have time to discuss the philosophy of reflex action in its relations to nervous phenomena, but will simply remark that most of the facts which I am about to present fully establish the importance of the knowledge that all morbid manifestations may be due to a reflex influence. I will only allude, in passing, to one or two facts in connexion with this part of my subject, which would be observable in the relation of cause and effect by the most casual observer. The fact that a wound of a superficial part of the body produces tetanus, is a well known circumstance, and in itself is a sufficient *a priori* evidence that the cause of that nervous affection may proceed from a reflex influence. Again, the fact that epilepsy is induced in some instances by the pressure of a bullet upon a branch or the trunk of a nerve, and is as quickly and effectually cured by the removal of the missile is a very patent argument in favor of the theory. I do not think it necessary to multiply examples; but will make the statement that in almost all nervous affections, whether functional or organic, the cause is very often a reflex influence. For instance, a marked congestion of the spinal cord, or of the brain itself, can very frequently be traced to a peripheric irritation, as its starting-point. Let a cold current of air be applied to a part of the body that may be warm, the nerves of that particular part are irritated thereby, the irritation is transmitted to the brain or spinal cord, and congestion is the result. A full knowledge of the frequent production of nervous affections by a peripheric irritation, gives us a very important indication for treatment, which must necessarily consist in the removal of the cause of irritation, whatever it may be. If we can trace a congestion to cold applied to any particular part of the body, we must take the necessary means to counteract the bad influence which it has exerted upon the nervous centres by remedies applied directly to the starting-point of irritation. For instance, if the nervous symptoms can be traced to the immersion of the feet in cold water, we must direct our applications to these extremities; we must induce a counter-irritation of those parts. By this intelligent direction of our therapeutic means, we accomplish a great deal, and may even by the simplest remedies arrest what may seem to be, and if neglected may certainly become, a very serious disease.

After these few remarks, I come to another very frequent cause of nervous affections. This is also of very great importance to consider. Any alteration in the secretion of a gland, no matter how unimportant that organ may seem to be for the preservation of our health, may be the cause of a nervous complaint. I can give illustrations of this, were it not patent to the minds of every one that such is the case. In connexion with this point, I cannot urge upon you too strongly the necessity in every case of nervous affections of looking carefully after the condition of every organ of the body, and assuring yourselves, if possible, that no peripheric irritation exists. Such an irritation might be found to reside in an old cicatrix which has been healed twenty years, and in which not even pain is felt.

I pass now to consider a great many means of treatment which are of importance in nervous complaints.

*Pressure on the Carotid for Congestion of the Brain.*—I will first speak of pressure upon the carotid as a means which has been employed against headache, against congestion, and against inflammation of the brain. This treatment was employed in the view, that by stopping circulation in that blood-vessel, the amount of blood in the brain was diminished. But this is far from being the case. Whatever apparent good effect there may be from pressure in the region of the carotid, it has nothing to do in diminishing the supply of blood to the brain; but it is chiefly a pressure on the cervical sympathetic nerve which brings about the result; for that nerve being irritated by such pressure, causes a contraction of the blood-vessels of the brain.

*Ligature of the Carotid.*—The carotid has been tied for the cure of epilepsy; but this is a most irrational treatment, and I hope it will eventually be abandoned. It has been employed not only in epilepsy, but in diseases of the brain, which, in common with that affection, were supposed to depend on congestion of the brain. But in this case, as in the one in which simple pressure on the carotid is used, the congestion is diminished by some irritation of the sympathetic. If surgery should be bold enough to divide this nerve (which, perhaps, may be before long,) a great advance might be made in the treatment of epilepsy. The loss of consciousness depends altogether, in the beginning of the attack, on the contraction of the blood-vessels of the cerebral lobes, producing in these parts of the brain a state of syncope. The loss of consciousness which occurs in the *petit mal*, might be avoided altogether by the extirpation of an inch or an inch and a half of the cervical sympathetic nerve. The teachings of physiology and pathology, and particularly the results of my experiments on animals rendered epileptic, conclusively show that there can be no chance for a loss of consciousness after this operation is performed.

*Cauterization of the Urethra.*—Lallemand, who has written an excellent work on seminal losses, and the nervous complaints due to this genital affection, has employed against it a plan of treatment which consists in cauterization of the urethra. There is a much better mode of treatment, I think, for these complaints than the severe and rather dangerous one of cauterization, which not rarely causes a stricture. The means which I have found most available are: Pressure on the prostate, whereby the congestive state of that organ is diminished. The same thing will happen when ice or a very cold douche is applied to the perineum. I have also often obtained a considerable amelioration, and sometimes a cure, by a medicinal and hygienic plan of treatment, consisting in the use of atropine, the ergot of rye, large doses of the bromide of potassium, with tonics. Atropine and the bromide of potassium have a specific action on the prostate and the genital organs; and so great is that power with the bromide, that it will reduce the pain and erection in chordee, and diminish if not abolish sexual desire. But the dose of the bromide, to produce these effects, must be a large one; for instance, a drachm twice a day. There is no danger in using it in such quantities, and the only effect which may be troublesome is that the patient may get very sleepy.

*Tracheotomy*, etc.—Tracheotomy has been proposed by Marshall Hall against epilepsy. But the spasm of the glottis for which the operation is advised is only a symptom—a result, and not the cause of epilepsy. It is, then, only of service as a temporary amelioration. I need not say here that the theory of Marshall Hall in reference to epilepsy is, in my opinion, entirely wrong. However, it may be well to be prepared to operate if an emergency should arise. Dr. Lalor, of Dublin, has ascertained that food is sometimes thrown from the stomach into the larynx and trachea in fits of epilepsy, causing death by asphyxia. Tracheotomy would, of course, be useful in such cases.

*Extirpation of the Clitoris.*—Extirpation of the clitoris for nervous affections is an operation which cannot be approved of on rational grounds. Mr. Baker Brown has been in the habit of treating several kinds of nervous affections by this method; but I cannot agree with him in thinking that it is a proper treatment, when there is not a manifest connexion between a morbid state of that organ and the nervous disorders. You know that onanism is a very frequent cause of nervous affections in women as well as men. There are cases on record, and others not published, in which that operation has not been attended either with a good effect upon the nervous trouble or upon the loathsome habit. I know several cases of this kind. That which I have found more serviceable when moral advice has failed, is the production of a sore close upon one of the parts handled, so that every attempt at friction will cause a pain compelling the patient to abstain from it. The sore must be kept open until the general health is sufficiently restored to allow the moral influences to be brought to bear upon the patient with good effect.

*Extirpation of the Testicle.*—As regards ablation of the testicles, I cannot speak in words of disapproval strong enough. It seems to be not only a very irrational but a barbarous procedure, and it was certainly so in most of the cases in which this operation was performed against epilepsy.

*Section of Nerves.*—One of the modes of treatment which have been found of great importance in the treatment of epilepsy, tetanus, and other complaints which are due to an irritation of nerves by a wound, a burn, etc., consists in the section of irritated nerves, at some distance above the wound or burn—*i. e.*, between the point of irritation and the nervous centre.

*Actual Cautery.*—In regard to the use of actual cautery in nervous affections, functional and organic, I am sorry to say that this method of treatment, which is perhaps too much employed in France, is too much neglected in England and in this country. I have often applied the white-hot iron to the head of patients in the coma of apoplexy, of cerebritis, of uræmia, or of epilepsy, with almost always the good effect of freer breathing. Many patients have been aroused from coma by that means alone, and not a few have recovered, whose recovery was at least partly due to that mode of treatment. The application of the hot iron to the nape of the neck has been often found of signal service, in cases of any functional nervous complaint in which it may be found necessary to change the nutrition at the base of the brain. I hardly need say that the actual cautery is one of the best, if not the best means of treatment of neuralgia and rheumatic pains.

*Application of Ice.*—Opposite to this, but acting in the same way, is the local application of ice to the spine and various parts of the body. This plan of treatment has also been too much neglected. It may be extremely beneficial in a number of affections. Dr. Chapman, of London, has published some cases in which the beneficial results of the application of ice to the spine in certain nervous complaints have been very apparent. These cases are not so complete as one would wish to have them; still I have no doubt that the principle upon which he founds his plan of treatment is a tenable one. I have no doubt also, that ice to the spine in certain kinds of epilepsy, particularly in those cases where there is evidence of a spinal irritation, will be of real benefit.

*Circular Blisters and Ligatures.*—I come now to another method of treatment, which, according to the results of my own practice, is of considerable importance. When I began to have charge of an immense number of paralytics and epileptics at the National Hospital in London, I found that, in most of the cases in which ligatures are useful against epilepsy, they act in quite a different way than that which is generally admitted. I ascertained that it is the irritation of the nerves of the skin, and not a pretended obstacle to the passage of the so-called aura, which, in cases of an aura of centric origin, produces the good effects of preventing a fit or diminishing its intensity. I found that pinching, pricking, or a blow, acted almost as well as a ligature; and these facts, and others that I have not time to mention, led me to admit that an irritation of the nerves of the skin in limbs can produce a very favorable change in the state of the base of the brain, and also in the spinal cord. This view made me adopt a plan of treatment which has proved useful, not only in epilepsy, but in hysteria, in chorea, in trembling palsy, and in cases of congestion or inflammation of the base of the brain, or in cerebral meninges. This plan consists in the application of blisters, one inch in width, all round a limb attacked with either paralysis, trembling, jerks, or pain, due to a congestion or inflammation of the nervous centres or their meninges. These circular blisters produce an irritation of cutaneous nerves through which a favorable change takes place in the circulation or nutrition of the nervous centres. This mode of treatment is generally much more useful than the application of counter-irritation to the head, the nape of the neck, or the spine.

*Errhines.*—There is another means of treatment which I cannot pass by without a notice, and that is the use of errhines. I am convinced that these are not employed as often as they ought to be. These medicaments are of special utility in cases of nervous affections (epilepsy, headache, congestion of the brain) attended with sensations of fulness, heaviness, tightness, etc., in the frontal sinuses.

Within the last few years subcutaneous injections of morphine, quinine, etc., have been very much employed. I will only say that this plan of treatment is a most invaluable one, and that it is absolutely free from any great danger if carefully employed. There is frequently, however, *some* danger, and medical men should be prepared for it. I do not mean to say that life is compromised by the treatment, but that phenomena will sometimes occur which may seriously compromise the reputation of the practitioner. I usually employ morphia, together with opium, in cases in which it seems useful to inject narcotics. The doses are from one-third to one-half of a grain of the sulphate of morphine, with from the one-sixtieth to the one-fortieth of a grain of the sulphate of atropine. I have for a good many years injected these narcotic salts together. Not that I believe that they are antagonistical in the way that many suppose. I deny that a dose of morphine, able to destroy life, can be neutralized by any quantity of atropine. I have an opinion contrary to authorities in this respect. I have given a dose, by injection, to a dog sufficient to kill him in a certain time, and have immediately afterwards injected the prescribed amount of atropine to counteract it. I have seen most of the symptoms caused by the first alkaloid diminish in consequence of the action of the last injection, but death took place nearly at the same time as if I had not injected the atropine. My experiments prove that these narcotic remedies and poisons are not antagonistic as regards their deleterious, toxic, or poisonous effects, however much they are antagonistic as regards some of their effects on the nervous centres, the eyes, etc. They should be employed together, according to my notion, on account of their real antagonism, and also on account of the addition of the good effects of each of them to those of the other, against pain particularly.

I now pass to consider other remedies. The bromide of potassium is an invaluable remedy. The dose for epilepsy should be a scruple three times a day. The best time to give it is when the stomach is empty, so as to allow of its rapid absorption. But I have found that, in certain forms of epilepsy, the use of bromide of potassium alone is not so good as when it is combined with other remedies. The best remedies to be used, together with this salt, are the iodide of potassium and the bromide of ammonium. The iodide of potassium will not do much alone against epilepsy, unless it be due to syphilis, and then the dose must be extremely large; but if the bromide of potassium be added to a small dose of the iodide, the good effect of these two salts employed together will be increased, especially in cases of epilepsy allied with, or due to, a congestion in the base of the brain, manifesting itself either by a slight hemiplegia, or by other symptoms. These two remedies are very different in their action, although there are many physicians who believe that they are almost identical. Bromide of potassium produces anæsthesia of the throat and larynx, dilatation of the pupil, deafness, anæsthesia of the urethra, and a diminution of the sexual power, etc.; but none of these symptoms are produced by the iodide of potassium. On the other hand, the iodide of potassium will sometimes cure syphilis, while the bromide of potassium has no power whatever over that disease.

I said bromide of ammonium should be sometimes employed together with the bromide of potassium. There are cases of epilepsy in which the combined use of these remedies is of great value. In cases where there is suspicion of the existence of congestion of the base of the brain, and still more of a congestion of the spinal cord, or its meninges, the bromide of ammonium will give a decidedly greater effectiveness to the bromide of potassium.

The bromide of ammonium is a very valuable remedy in whooping-cough, as first shown by Dr. Gibb and Dr. Harley, of London. As I have named that disease, I may here mention that there are other modes of treatment of more value. One of these modes is by the use of atropine. But if you employ it you have to be by your patient, as it is necessary to give it in doses large enough to produce delirium, and to keep up that condition for three days, except at night. In the evening you may give morphine, or what is better, codeine, or narceine. This will procure a quiet night's rest. You must watch your patient to see that the delirium does not go further than it ought to. If you can get the consent of the parents to allow the use of this remedy, you may be able to cure your patient at the end of three days, as I did in a bad case of whooping-cough in my own child. I need not say that the bronchitis, allied with the whoop, will persist some time after the cessation of this nervous phenomenon. It will, however, be very hard work to get the consent of the parents if you tell them beforehand how the medicine will act; but some may give you a chance.

I will now say a few words on another remedy, which is too little employed; I mean the chloride of barium. I have given it in one case of trembling palsy, with a very gratifying result. This is the only case of that affection in which I have obtained a complete cure. In some of the other cases in which I have tried that remedy, there was a marked amelioration of the symptoms. The dose of this remedy must be a large one—from half a grain to one grain three times a day. This is a dose which, according to writers on toxicology, might be sufficient to kill outright. I can only say that these writers are quite wrong. This remedy, in very large doses, has lately been employed with success in several cases of tetanus.

Codeine and narceine are remedies which I have used a great deal; more especially is this the case with the former. Codeine is a most valuable remedy to give a quiet night's rest. In this connexion I must refer again to the bromide of potassium as an invaluable remedy to produce sleep in persons who are wakeful on account of severe anxiety, grief, or any other moral cause. The dose too of this medicine must be pretty large. About four or five P. M. as much as thirty or forty grains, and another dose equally as large by eight or nine in the same evening.

I will only say a few words on two or three other remedies. In nervous affections, due to congestion of the kidneys, or of the ovaries, I have found the use of gallic acid in four or five grain doses, six times a day, of singular efficacy. It is much better to give it in frequently repeated doses than otherwise, as it is less apt to disturb the stomach.

The nitrate of silver has been considered a very excellent remedy in the treatment of that newly-studied affection, the locomotor ataxy. The great expectations that were entertained of its efficacy have not, however, been realized, and it has been found that instead of curing the majority of the cases, it has sometimes only succeeded in blackening the skin. It is, however, when used properly, of some value; but it should not be used longer than a month at a time, for by allowing an intermission, say of a month, it has pretty much as good an effect as when taken without interruption, and does not alter the color of the skin.

In conclusion, gentlemen, allow me to make this single remark, that in the treatment of nervous affections, you cannot search too diligently for their cause in all the organs of the body; and that far from the study being an exclusive and especial one, it is necessary, before undertaking to practise with chances of success in this department of the medical art, to have a knowledge of the diseases of every organ. The best specialist for nervous affections is he who joins to a specially extensive knowledge of all the forms and varieties of nervous complaints, a thorough acquaintance with all the general and special diseases and disorders of every part of the human frame in themselves, and particularly in their relations with the nervous system.—*N. Y. Medical Record.*

